# *Burkholderia pseudomallei *genome plasticity associated with genomic island variation

**DOI:** 10.1186/1471-2164-9-190

**Published:** 2008-04-25

**Authors:** Sarinna Tumapa, Matthew TG Holden, Mongkol Vesaratchavest, Vanaporn Wuthiekanun, Direk Limmathurotsakul, Wirongrong Chierakul, Edward J Feil, Bart J Currie, Nicholas PJ Day, William C Nierman, Sharon J Peacock

**Affiliations:** 1 Mahidol-Oxford Tropical Medicine Research Unit, Faculty of Tropical Medicine, Mahidol University, Bangkok, Thailand; 2 The Wellcome Trust Sanger Institute, Hinxton, Cambridge, UK; 3 Department of Biology and Biochemistry, University of Bath, UK; 4 Menzies School of Health Research, Charles Darwin University, Darwin, Australia; 5 Center for Clinical Vaccinology and Tropical Medicine, Nuffield Department of Clinical Medicine, University of Oxford, Churchill Hospital, Oxford, UK; 6 J. Craig Venter Institute, Rockville, Maryland 20850, USA; 7 The George Washington University School of Medicine, Department of Biochemistry and Molecular Biology, Washington DC 20037, USA

## Abstract

**Background:**

*Burkholderia pseudomallei *is a soil-dwelling saprophyte and the cause of melioidosis. Horizontal gene transfer contributes to the genetic diversity of this pathogen and may be an important determinant of virulence potential. The genome contains genomic island (GI) regions that encode a broad array of functions. Although there is some evidence for the variable distribution of genomic islands in *B. pseudomallei *isolates, little is known about the extent of variation between related strains or their association with disease or environmental survival.

**Results:**

Five islands from *B. pseudomallei *strain K96243 were chosen as representatives of different types of genomic islands present in this strain, and their presence investigated in other *B. pseudomallei*. *In silico *analysis of 10 *B. pseudomallei *genome sequences provided evidence for the variable presence of these regions, together with micro-evolutionary changes that generate GI diversity. The diversity of GIs in 186 isolates from NE Thailand (83 environmental and 103 clinical isolates) was investigated using multiplex PCR screening. The proportion of all isolates positive by PCR ranged from 12% for a prophage-like island (GI 9), to 76% for a metabolic island (GI 16). The presence of each of the five GIs did not differ between environmental and disease-associated isolates (p > 0.05 for all five islands). The cumulative number of GIs per isolate for the 186 isolates ranged from 0 to 5 (median 2, IQR 1 to 3). The distribution of cumulative GI number did not differ between environmental and disease-associated isolates (p = 0.27). The presence of GIs was defined for the three largest clones in this collection (each defined as a single sequence type, ST, by multilocus sequence typing); these were ST 70 (n = 15 isolates), ST 54 (n = 11), and ST 167 (n = 9). The rapid loss and/or acquisition of gene islands was observed within individual clones. Comparisons were drawn between isolates obtained from the environment and from patients with melioidosis in order to examine the role of genomic islands in virulence and clinical associations. There was no reproducible association between the individual or cumulative presence of five GIs and a range of clinical features in 103 patients with melioidosis.

**Conclusion:**

Horizontal gene transfer of mobile genetic elements can rapidly alter the gene repertoire of *B. pseudomallei*. This study confirms the utility of a range of approaches in defining the presence and significance of genomic variation in natural populations of *B. pseudomallei*.

## Background

*Burkholderia pseudomallei *is a soil dwelling Gram-negative bacterium and the cause of melioidosis, a serious human infection commonly reported in northeast Thailand and northern Australia [1,2]. The genome of this biothreat agent contains genomic island (GI) regions that have properties indicative of horizontal gene transfer, i.e. DNA displaying anomalies in % G+C content or dinucleotide frequency signature, and/or the presence of coding sequences (CDSs) with similarities to genes associated with mobile genetic elements such as insertion sequence (IS) elements, bacteriophages and plasmids. Sixteen GIs were identified in the genome of *B. pseudomallei *strain K96243 [3], comprising approximately 6% of the genome. Screening of 40 clinical and environmental isolates of *B. pseudomallei *for the presence of selected K96243 GIs found that there was variable distribution [3], suggesting that horizontal gene transfer contributes to genome diversity. Genomic islands in other bacterial species encode many different functions, and selection may favour the maintenance of islands that increase fitness in a specialized environmental niche. Genomic islands also play a pivotal role in virulence of a large number of bacterial pathogens, carrying clusters of virulence genes encoding a wide range of functions including iron uptake systems, adhesins, superantigens, and genes that alter the antibiotic resistance phenotype [4].

*B. pseudomallei *is found in the soil and surface water of endemic areas and usually enters the host via cuts and abrasions in the skin or by inhalation following contact with contaminated water or soil. Once inside the body, the time taken for disease symptoms to appear can vary from hours to years. In some cases, *B. pseudomallei *infection does not cause overt disease around the time of exposure, but rather results in a state of bacterial dormancy in the host for many years before causing disease in later life. The most remarkable example is that of a second world war veteran who developed melioidosis 62 years after returning to the US from SE Asia [5].

The clinical symptoms of melioidosis are so varied that the disease has been termed "the great mimicker" [6]. Disease manifestations are extremely wide ranging and vary from acute, fulminant sepsis to localized disease. The most frequent picture is a septicemic illness, often associated with bacterial dissemination to distant sites such as the liver and spleen [2]. Pneumonia occurs in around 50% of patients. Other sites of infection include bone, joints, skin, soft tissue, prostate and the central nervous system. Overall mortality is around 50% in northeast Thailand (35% in children) [2], and 19% in Australia [1]. The basis for marked variability in clinical presentation and disease severity is unknown. Host determinants such as diabetes mellitus and renal failure represent risk factors for infection with *B. pseudomallei *[1,7], but no host factors have been associated with specific clinical features. The role of bacterial factors in determining disease variability and severity is poorly understood.

In this study we examined the distribution of a representative sample of five GIs previously described for *B. pseudomallei *K96243. *In silico *analysis of 10 genome sequences revealed the micro-evolutionary processes responsible for the short-term diversification of these regions. Multiplex PCR screening was used to investigate GI presence in *B. pseudomallei *isolates from NE Thailand. Rapid loss and/or acquisition of gene islands was observed by assaying the presence of islands within isolates belonging to single clones (as defined by multilocus sequence typing (MLST)) [8]. Comparisons were drawn between isolates obtained from the environment and from patients with melioidosis in order to examine the role of genomic islands in virulence.

## Results and Discussion

### Overview of five genomic islands in *B. pseudomallei *strain K96243

The five genomic islands studied here include examples of a prophage (GI 2; φK96243) which has been shown to be mobile in this strain, two prophage-like islands (GI 6 and GI 9), a putative integrated plasmid (GI 11), and a putative metabolic island (GI 16) that lacks any obvious genes for mobilisation or integration [3]. The five islands are a representative sample of the islands present in a single strain, and provide a snapshot of GI diversity. The mosaic structure and variable composition of GIs means that it is impractical to attempt to capture the full gamut of elements circulating in a large natural bacterial population, and we therefore focused our analysis on a representative sample of GIs identified in K96243.

A summary of the CDS content of each of the five GIs in *B. pseudomallei *K96243 is provided in Additional file 1, and a comprehensive list of the CDSs contained in these regions is provided in Additional File 2. The overwhelming majority of CDSs that are not phage or plasmid related are of unknown function. GIs 2, 6, 9 and 11 contain CDSs with no homology to known metabolic or putative virulence proteins. GIs 2, 6 and 11 contain low % GC regions relative to the rest of the island, indicating that the islands are mosaic and some genes may have been acquired recently; new gene acquisitions may have atypical sequence characteristics that are maintained over a relatively short time frame. GI 16 contains several CDSs with similarity to known virulence determinants, including a putative haemagglutinin and processing protein, and genes involved in the acquisition and utilization of nutrients. The abundance of CDSs on this island that encode functions that potentially broaden the metabolic repertoire of K96243 (for example possible sugar utilization and amino acid catabolism gene clusters and accompanying regulators), has led to this island being previously designated a putative metabolic island [3]. CDSs in this island region exhibited varying levels of similarity to proteins from a range of taxonomically diverse organisms, and it was not possible to speculate on the likely source of this island region from similarity searches.

### Comparative genomic analysis of genomic islands

*In silico *analysis of the five GIs was undertaken to explore the degree of variability in presence, structure and chromosomal insertion site. Nine isolates that have been sequenced or are currently undergoing whole genome sequencing by JCVI (J. Craig Venter Institute) were examined and compared with strain K96243, which has been sequenced by the Wellcome Trust Sanger Institute [3] (Table 1). Seven JCVI isolates and K96243 are unique strains, but strains 1106b and 1710b were recovered from patients presenting with recurrent melioidosis due to relapse (failure to eradicate the primary infection), and are identical by PFGE and MLST to strains 1106a and 1710a, respectively.

**Table 1:** Distribution of islands in sequenced *B. pseudomallei *strains

**BP strain**	**Origin**	**GI 2**	**GI 6**	**GI 9**	**GI 11**	**GI 16**
K96243	Thailand	+	+	+	+	+
406e	Thailand	-	-	-	-	+/- (BPSS2060 to BPSS2076)
1106a†	Thailand	-	-	-*	Alt	+/- (BPSS2057 to BPSS2076)
1106b†	Thailand	-	-	-*	Alt	+/- (BPSS2057 to BPSS2076)
1710a†	Thailand	-	-	-	-	+/- (BPSS2057 to BPSS2076)
1710b†	Thailand	-	-	-	-	+/- (BPSS2057 to BPSS2076)
Pasteur 6068	Vietnam	+	-	-	-	+/- (BPSS2057 to BPSS2076)
S13	Singapore	+	-	-	-	+/- (BPSS2057 to BPSS2076)
668	Australia	-	-	-	-	-
1655	Australia	- *	-	-	-	+ *

† Strain pairs 1106a & 1106b, and 1710a and 1710b were obtained from two patients who each relapsed (from a persistent focus) 3 years after the primary episode of melioidosis. 'a' denotes primary isolate and 'b' denotes relapse isolate.

- Island absent; + Island present; +/- Island partially present (CDSs missing); -* Island absent at the site of insertion but similar region present elsewhere in the genome; +* Island present and contains extra sequence; Alt Alternative island present at this site (no sequence similarity)

We examined the structure, composition and insertion site of the five GIs using the ten available genome sequences (complete and assemblies from unfinished genomes). GIs 2, 6, 9 and 11 all had flanking repeats, integrases, and mobile element-like genes, consistent with the suggestion that these were recently acquired mobile genetic elements. There were two instances where the GI was absent at the predicted site of insertion, but DNA with extended similarity to the island was detected elsewhere in the genome (one example each for GI 2 and GI 9). In *B. pseudomallei *strain 1655 a prophage with extended similarity to GI 2 (95–99% DNA identity over ~95% of the length of GI 2; data not shown), is integrated at an alternative tRNA gene on chromosome 1 (tRNA-Arg as opposed to tRNA-Phe). In the genome of K96243, GI 12 is found at this alternative locus. In 1106a an island with similarity to GI 2 is inserted at an alternative locus (tRNA-Ser) on chromosome 1. An identical island is present in 1106b. The 1106a island has a mosaic structure; approximately half of the island is highly similar (95–99% DNA identity) to the K96243 GI 9, with the other half exhibiting no similarity (see Additional file 3). The location of these sequences at alternative loci may be due to genomic rearrangement following divergence from a common ancestor carrying the island, or may reflect the presence of multiple insertion sites for related mobile genetic elements in the *B. pseudomallei *genome.

**Figure 1:**
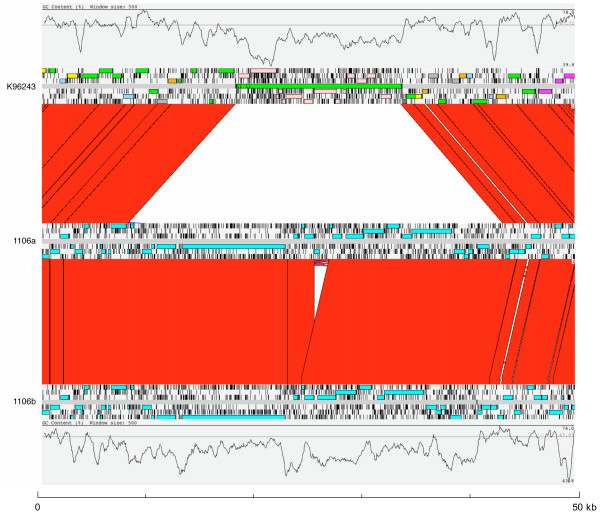
**Comparison of the GI 11 regions in *B. pseudomallei *strains K96243, 1106a and 1106b**. The results of a TBLASTX comparison of the GI 11 regions from strains K96243 (top) 1106a (middle) and 1106b (bottom) are displayed using the Artemis Comparison Tool (ACT) [21]. A plot of the % GC content of each sequence is displayed. The colored bars separating each genome (red and blue) represent similarity matches identified by BLASTN analysis [22]. Red lines link matches in the same orientation; blue lines link matches in the reverse orientation.

Comparative analysis of GI 11 region identified an alternative island inserted at orthologous sites in the 1106a and 1106b genomes (Table 1; Figure 1). This novel island is larger than the K96243 GI 11 (~35 kb). Comparison of the islands in 1106a and 1106b demonstrated that they are virtually identical except for a small internal indel region that is present in 1106a but absent in 1106b. The indel region is between two identical IS elements in 1106a. In 1106b there is a single insertion sequence (IS) element, suggesting that recombination between the IS elements may have led to the deletion in this region in 1106b. Although the 1106a and 1106b islands appear to be inserted at the same site as the K96243 GI 11, the attachment sites are different. In the case of K96243, the attachment site is an Ala tRNA, generating 45 mer perfect repeats that flank the island. The GI 11 regions in 1106a and 1106b are next to the Ala tRNA, but the flanking DNA in both islands is a 13 mer sequence that does not appear to be part of the Ala tRNA, or surrounding sequence in K96243. The alternative GI 11 in 1106a and 1106b contains a putative filamentous haemagglutinin and processing protein that are similar to proteins found in *B. thailandensis *(BTH_I2723 and BTH_I2721 respectively). The homologues of the filamentous haemagglutinin and processing protein in *B. thailandensis *strain E264 are not in an orthologous position relative to the *B. pseudomallei *GI 11 flanking DNA. In the genome of this closely related species, an alternative GI 11 is present (BTH_I3130 to BTH_I3143), which is integrated at the orthologous attachment site (Ala tRNA) to the GI 11 in *B. pseudomallei *K96243.

**Figure 2:**
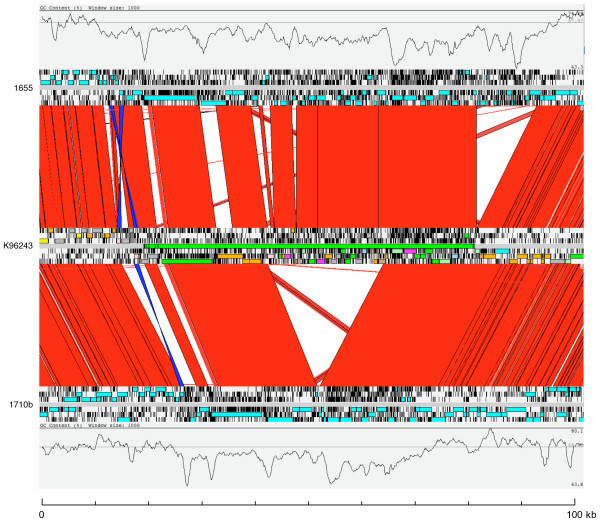
**Comparison of the GI 16 regions in *B. pseudomallei *strains 1655, K96243 and 1710b**. The results of a BLASTN comparison of the GI 16 regions from strains 1655 (top), K96243 (middle) and 1710b (bottom) are displayed using the Artemis Comparison Tool (ACT) [21]. A plot of the % GC content of each sequence is displayed. The colored bars separating each genome (red and blue) represent similarity matches identified by BLASTN analysis [22]. Red lines link matches in the same orientation; blue lines link matches in the reverse orientation.

Micro-evolutionary diversification was also observed in GI 16, in which an internal deletion encompassing CDSs BPSS2057 to BPSS2076 was noted in four unique strains and both relapse isolates (Table 1). Strain 406e also contains the island, but has a deletion encompassing the same region (BPSS2060 to BPSS2076) that has different boundaries, suggesting that the deletion occurred independently in this strain. One other strain (1655) contained the complete GI 16 plus additional sequence, both within the island and downstream of it. Figure 2 shows a comparison of GI 16 regions in strains K96243, 1655 and 1710b (which has the internal deletion of BPSS2057 to BPSS2076). The lack of integrases and other mobile genetic element CDSs, and high variability in CDS content, suggests that GI 16 is distinct from the other GIs considered here which have structures more typical of mobile genetic elements. GI 16 has a mosaic structure consistent with a long history of multiple insertion and deletion events. The role of horizontal gene transfer in the evolution of this region is supported by the anomalous GC composition (Figure 2).

### Presence of five genomic islands in *B. pseudomallei*

By assaying the presence of GIs in a large strain collection within the population framework provided by MLST data, it is possible to investigate the frequency of island acquisition and loss, as well as their possible role in disease. Presence or absence of the five GIs was defined for 186 Thai isolates obtained from the environment (n = 83) and from patients with melioidosis (n = 103). The proportion of all isolates positive by PCR varied between GIs, and ranged from 12% for a prophage-like island (GI 9) to 76% for a metabolic island (GI 16) (Table 2). These proportions are consistent with previous findings reported for 40 Thai isolates [3], with the exception that GI 2 and GI 6 were detected more frequently in the larger bacterial population examined here (72% versus 53% for GI 2, and 23% versus 8% for GI 6, p = 0.02 & p = 0.03, respectively). Isolates in both collections were from northeast Thailand. A small proportion of strains were positive for one or two gene targets for a given GI, and gave an amplification product for PCR across the putative insertion site (data not shown). One explanation for this is that GIs may have alternative insertion sites. This explanation was shown to be true for some islands on the comparative genomic analysis of the sequenced strains, described above.

**Table 2:** Detection by PCR of five genomic islands in 186 *B. pseudomallei *isolates from northeast Thailand

**Genomic island**	**Positive (%)**	**Positive (%)**		
		
	**All isolates (n = 186)**	**Soil (n = 83)**	**Invasive (n = 103)**	***P***^***a***^
2	133 (72%)	62 (75%)	71 (69%)	0.39
6	43 (23.%)	22 (27%)	21 (20%)	0.33
9	22 (12%)	11 (13%)	11 (11%)	0.59
11	39 (21%)	15 (18%)	24 (23%)	0.38
16	142 (76%)	66 (80%)	76 (74%)	0.36

**Cumulative number of genomic islands**	**All isolates**	**Soil**	**Invasive**	***P***^***b***^

0	5 (3%)	1 (1%)	4 (4%)	0.28
1	46 (25%)	20 (24%)	26 (25%)	
2	84 (45%)	36 (43%)	48 (47%)	
3	40 (22%)	20 (24%)	20 (19%)	
4	10 (5%)	6 (7%)	4 (4%)	
5	1 (0.5%)	0	1 (1.0%)	

^*a *^Chi-square test; ^*b *^Student's t test

### Variability in genomic islands between clones of *B. pseudomallei*

We explored the mobility of the five GIs by examining whether the presence of islands reflects the underlying population structure, such that closely related isolates might have more similar GI repertoires than distantly related isolates. This was possible as all of the isolates used in this study had been characterized previously by MLST [9]. Analysis was restricted to the three largest clones (ST 70 (n = 15 isolates, allelic profile (gene order *ace-gltB-gmhD-lepA-lipA-narK-ndh*) 3-4-11-3-5-4-6), ST 54 (n = 11, allelic profile 3-1-3-3-1-2-1), and ST 167 (n = 9, allelic profile 1-1-4-1-1-3-1)). Variation in the GI repertoire was defined within and between these clones. There was considerable variability in presence of the five GIs between the three clones (Table 3). For example, GI 6 was more common in isolates belonging to ST 70 than the other two clones (p = 0.04), GI 16 was ubiquitous in ST 54 isolates but present in three quarters or less of the other two STs (p = 0.04), and GI 11 was only detected in 4 isolates belonging to ST 70. An analysis of the difference in GIs between environmental and clinical isolates in each clone was not performed because of the lack of statistical power.

**Table 3:** Presence of genomic islands in the three largest clones as defined by multilocus sequence type

**Genomic island**	**All isolates**	**Sequence type**			
			
		**ST 70**	**ST 54**	**ST 167**	***P***^***a***^
Number of isolates	186	15	11	9	-
GI 2	133 (72%)	11 (73%)	6 (55%)	6 (67%)	0.60
GI 6	43 (23%)	8 (53%)	1 (9%)	1 (11%)	0.04
GI 9	22 (12%)	0	1 (9%)	0	0.57
GI 11	39 (21%)	4 (27%)	0	0	0.08
GI 16	143 (77%)	10 (67%)	11 (100%)	5 (56%)	0.04

^*a *^Fisher's exact test

These findings suggest that horizontal gene transfer can alter the gene repertoire ("accessory genome") of this species very rapidly, i.e. before any observable changes in the vertically inherited "core" genome, and is consistent with the hypothesis that isolates that are identical by MLST might exploit different ecological niches [10]. The MLST data for *B. pseudomallei *has revealed a very high frequency of homologous recombination resulting in limited clustering and low levels of linkage disequilibrium [8,11,12]. Our results also suggest that the loss and acquisition of genomic islands by horizontal gene transfer significantly contributes to the dynamism of the genome. However, it should be noted that these two processes are distinct, and that a high rate of island transfer does not necessarily predict a high rate of homologous recombination or *vice versa*. For example, MLST data for the Gram-positive pathogen *Staphylococcus aureus *has revealed only modest rates of homologous recombination, yet the "accessory" genome of this species is known to change very rapidly [10,13-16]. *B. pseudomallei *represents an alternative scenario, the data indicating both high rates of homologous recombination together with rapid changes in GI repertoire.

### Comparison of the presence of genomic islands in soil and invasive isolates

We examined the hypothesis that the GIs play a role in the ability to cause human disease by testing whether the presence of GIs was significantly different in clinical and environmental isolates. We found that there was no significant difference in the presence of any of the GIs between these two groups (p > 0.05 for all five islands) (Table 2). We considered the possibility that combinations of GIs could alter the ability to cause disease through either a cumulative or synergistic effect. We defined the cumulative number of GIs per isolate for the 186 Thai isolates; this ranged from 0 to 5 (median 2, IQR 1 to 3) (Table 2). The distribution of cumulative GI number did not differ between environmental and disease-associated isolates (p = 0.27) (Table 2). These data indicate that none of the islands examined were numerically associated either individually or on a cumulative basis with the ability to cause melioidosis.

### Relationship between clinical features and genomic islands in disease-associated isolates

The lack of association between the presence of the five GIs and the ability to cause human disease does not exclude the possibility that these GIs are associated with specific clinical features or outcome. To study this, we undertook a detailed statistical analysis of the relationship between a range of clinical features in 103 patients with melioidosis and the presence of GIs in their associated *B. pseudomallei *isolates. Univariate analysis was used to compare the presence of each GI and their cumulative number with the presence of blood cultures positive for *B. pseudomallei*, presence of ultrasound confirmed abscess(es) in liver and/or spleen, pneumonia, multiple organ involvement, the presence on admission or subsequent development of hypotension, impaired renal function, impaired liver function and death during hospital admission. GI 6 was negatively associated with positive blood culture (OR 0.30; 95%CI 0.11–0.82, p = 0.02), and GI 11 was negatively associated with impaired renal function (OR 0.36; 95%CI 0.14–0.94, p = 0.04). Cumulative number of GIs was negatively associated with impaired renal function (OR 0.54; 95%CI 0.33–0.86, p = 0.01). These significant associations could be a chance finding related to multiple comparisons. To test this hypothesis, the presence of GIs were determined for an independent set of 255 invasive isolates obtained from patients with melioidosis presenting to Sappasithiprasong Hospital between 2002–2003. There was no significant association between GI 6 and positive blood cultures (OR 1.12; 95%CI 0.51–2.43, p= 0.78), or GI 11 and impaired renal function (OR 0.67; 95%CI 0.34–1.32, p = 0.25). Cumulative number of GIs was not negatively associated with impaired renal function. We conclude that there were no significant associations between clinical features and any of the five GIs examined.

## Conclusion

This survey of divergent putative GIs in a well-defined and clinically significant *B. pseudomallei *population has demonstrated that genetic exchange is widespread. Our *in silico *analysis provided evidence that GI 16 may represent a composite region rather than a genomic island, and defined a novel GI at the predicted insertion site for GI 11. A detailed analysis of the relationship between five GIs and both the ability to cause disease and a range of clinical features failed to show an association between the two. However, a previous study that utilized MLST to examine the bacterial genotype of 266 *B. pseudomallei *isolates (83 soil and 183 invasive) obtained from northeast Thailand reported that isolates from patients with melioidosis were significantly over-represented in the 10 largest clones [9]. A classification index applied to examine differences in the frequency of STs, and the frequency of alleles at specific loci, also demonstrated a significant difference between the two bacterial populations. This indicates that bacterial genotypes were not distributed randomly between soil and invasive isolate groups, an observation that could be accounted for by variations in the accessory genome of some strains that leads to heightened virulence. Further evidence for this suggestion comes from a study in Australia, in which variability in genome content between two Australian *B. pseudomallei *isolates was utilized to define sequence variability and to develop a variable amplicon typing scheme designed to score the presence of 14 PCR amplicons in Thai and Australian isolates [17]. Isolates clustered into groups, one of which was mostly associated with severe disease.

Our study confirms the utility of a range of approaches in defining the presence and significance of genomic variation in natural populations of *B. pseudomallei*. Further studies are required to determine the full extent of variability in GIs, and their relationship to biological fitness in the environment and to disease pathogenesis in the human host.

## Methods

### Bacterial isolates

A total of 186 *B. pseudomallei *isolates were obtained from northeast Thailand. Of these, 83 were isolated from the environment in the province of Ubon Ratchathani, northeast Thailand between 1990 and 2003. All sites were flooded rice paddies, the majority of which were sampled after ploughing but before planting. Environmental samples were processed for the presence of *B. pseudomallei*, as previously described [18]. A further 103 isolates were cultured from consecutive patients presenting with melioidosis during 2001 to Sappasithiprasong hospital, Ubon Ratchathani. Clinical manifestations of infection were varied; 63 patients had positive blood cultures with or without involvement of one or more organs or tissues, and 40 patients had negative blood cultures but one or more organs or tissues involved. All isolates were maintained at -70°C in TSB with 15% glycerol.

### Detection of genomic islands

A single bacterial colony was inoculated into TSB and incubated overnight in air at 37°C, after which genomic DNA was extracted using the Wizard Genomic DNA purification kit (Promega, WI, USA). The presence or absence of five GIs (GI 2, prophage φK96243; GI 6, prophage-like; GI 9, prophage-like; GI 11, putative integrated plasmid; and GI 16, metabolic island) [3], was defined by PCR. Two target genes were selected for each island, as follows: BPSL0130 and BPSL0135 (GI 2), BPSL 1138 and BPSL1155 (GI 6), BPSL2578 and BPSL2579 (GI 9), BPSL3258 and BPSL3260 (GI 11), and BPSS2053 and BPSS2061 (GI 16). Targets encoded predominantly putative hypothetical proteins with unknown functions. Genes that encoded functions necessary for the transfer and maintenance of the island such as integrases were avoided as similar sequences may be found on other unrelated islands. Primers and cycling conditions are as described by Holden *et al*. [3] with the exception of the following: BPSL1030-f (5'-GCGCCGCTCGACTTCCTTCTCT), BPSL1030-r (5'-GAGGGGCCGGACTGCTACTTCAC); BPSL1138-f (5'-GATTTGGTTGGCGTCCGTGTTT), BPSL1138-r (5'-CGACCTTGGCCGAATTATGTGA); BPSS2061-f (5'-AACGCTCGCGCCCTTTAC), BPSS2061-r (5'-AATGCCCTTCCGAATCCTTTATG). A positive control (*B. pseudomallei *K96243) and a negative control (reaction mixture without DNA) were included in each PCR run. To verify the absence of the genomic islands, a second round of PCR was performed for all isolates to amplify the putative insertion site by using primers situated within genes immediately abutting each putative island, as previously described [3]. The expected result was either no PCR product (interpreted as being consistent with the presence of the island) or a product whose size was consistent with amplification across the insertion site minus island PCR. Amplifications were performed using a PTC-0200 DNA engine (MJ Research, Cambridge, MA) with *Taq *polymerase (Promega), and aliquots of reaction mixtures were analyzed by 1% agarose gel electrophoresis. An island was classified as present if either or both target genes were positive, and negative if no amplification products were obtained for either target gene but a product was amplified across the putative insertion site.

### Clinical definitions

Multiple organ involvement was defined when there was >1 non-contiguous focus of infection, not including blood. Pneumonia was defined as the presence of clinical features plus radiographic changes and/or sputum culture positive for *B. pseudomallei*. Impaired renal function was defined as an estimated glomerular filtration rate below 60 mL/min/1.73 m^2 ^during admission. Glomerular filtration rate was estimated using an abbreviated form of the Modification of Diet in Renal Disease study equation [19]. Impaired hepatic function was defined as an elevation of aminotransferase more than 5 times the upper normal limit, or the presence of jaundice during admission. Hypotension was defined as a systolic BP <90 mmHg on or during admission.

### Analysis

MLST has been performed by us for all of the Thai isolates used in this study [9], MLST profiles for which are maintained on the *B. pseudomallei *MLST web site [20]. Genomic islands were previously identified in the genome of *B. pseudomallei *K96243 (accession numbers BX571965 and BX571966) [3]. Comparison of genome sequences was performed with ACT (Artemis Comparison Tool) [21] using BLASTN and TBLASTX [22]. Comparisons were made using the 3 other complete *B. pseudomallei *genome sequences 1106a (accession numbers CP000572 and CP000573), 1710b (accession numbers CP000124 and CP000125) and 668 (accession numbers CP000570 and CP000571), the 6 *B. pseudomallei *strains currently undergoing whole genome sequencing (406e, 1106b, 1710a, Pasteur 6068, S13, and 1655) by JCVI [23], and *B. thailandensis *E264 (accession numbers CP000086 and CP000085) [24].

Statistical tests were performed using the statistical program STATA/SE, version 9.0 (StataCorp LP, College Station, Tx.). Proportions were compared using the Chi-square test, or Fisher's exact test where appropriate. Comparison of continuous data was performed using the Student's t test. Unifactorial analysis was used to examine the association between presence of each GI, cumulative number of GIs and clinical factors.

## Authors' contributions

ST and MV carried out the laboratory aspects of the study (DNA extraction and PCR), MTGH carried out the bioinformatics component and drafted the manuscript, EJF and NPJD contributed towards study design and analysis and undertook critical review of the manuscript, VW, DK, WC and BJC contributed towards study design and undertook critical review of the manuscript, WCN assisted with the bioinformatics component and assisted with drafting the manuscript, and SJP conceived of the study, participated in the study design and coordination and drafted the manuscript. All authors have read and approved the final manuscript.

## Supplementary Material

Additional file 1 Overview of gene content within five genomic islands of *B. pseudomallei *K96243. The data provide an overview of gene content within five genomic islands of *B. pseudomallei *K96243 Click here for file

Additional file 2 Gene contents of five genomic islands. The data give a complete listing of the gene contents of five genomic islands of *B. pseudomallei *K96243 Click here for file

Additional file 3 Comparison of GI 9 in *B. pseudomallei *strain K96243 with a similar island in *B. pseudomallei *strain 1106a. The result of a BLASTN comparison of the GI 9 from K96243 (top) with a similar island present at an alternative locus in 1106a (bottom) is displayed using the Artemis Comparison Tool (ACT). The red bars represent similarity matches identified by BLASTN analysis. Horizontal green bars mark the extent of the island in each sequence. Click here for file
