# Supplementary material for: Burkholderia pseudomallei genome plasticity associated with genomic island variation
Source: BMC Genomics. 2008 Apr 25;9:190. doi: 10.1186/1471-2164-9-190 (PMC2386483; doi:10.1186/1471-2164-9-190)
Supplement: Additional file 1 — Overview of gene content within five genomic islands of B. pseudomallei K96243. The data provide an overview of gene content within five genomic islands of B. pseudomallei K96243 [file 1471-2164-9-190-S1.doc]

## Additional File 1 - Overview of gene content within five genomic islands of *B. pseudomallei* K96243

| **Island** | **Gene classification** | **No. CDSs** | **Functional Notes** |
| --- | --- | --- | --- |
| GI 2 | unknown function | 27 | 3’ region of the prophage contains a predicted membrane protein with no database matches in a low %GC region: BPSL0176 (55.9% GC) |
|  | bacteriophage structural and regulatory proteins | 22 |
| GI 6 | unknown function | 16 | 5’ region of the island contains two CDSs in a low %GC region: BPSL1137 (45.7% GC) and BPSL1138 (46.5% GC) that encode a putative nucleotide binding protein and conserved hypothetical protein with a RelA / SpoT conserved domain. |
|  | bacteriophage-related proteins | 5 |
|  | additional functional proteins | 1 |
| GI 9 | unknown function | 17 | Bacteriophage like-proteins include an integrase and a phage-like regulatory protein |
|  | bacteriophage-related proteins | 2 |
| GI 11 | unknown function | 7 | Plasmid like-proteins include conjugal transfer proteins and plasmid replication protein |
|  | plasmid-related proteins | 7 |
| GI 16 | unknown function | 11 | This island encodes several metabolic and transport functions that may be important in the generation and acquisition of nutrients. This includes two ABC transporter type systems, and a L-asparaginase, fatty aldehyde dehydrogenase, and putative alpha-galactosidase. The island also includes a putative cell surface haemagglutinin protein and two-partner secretion system protein that may have a role in niche colonization or virulence. |
|  | transport proteins | 8 |
|  | metabolic proteins | 6 |
|  | regulatory proteins | 2 |
|  | transposase proteins | 3 |
|  | misc. functional proteins | 8 |
|  | pseudogenes and gene remnants | 4 |
