# Supplementary material for: Burkholderia pseudomallei genome plasticity associated with genomic island variation
Source: BMC Genomics. 2008 Apr 25;9:190. doi: 10.1186/1471-2164-9-190 (PMC2386483; doi:10.1186/1471-2164-9-190)
Supplement: Additional file 2 — Gene contents of five genomic islands. The data give a complete listing of the gene contents of five genomic islands of B. pseudomallei K96243 [file 1471-2164-9-190-S2.doc]

**Additional file 2**

Gene contents of five genomic islands

| **ID** | **Predicted product** |
| --- | --- |
| **GI 2** | |
| BPSL0129 | prophage integrase |
| BPSL0130 | conserved hypothetical phage protein |
| BPSL0130a | hypothetical phage protein |
| BPSL0131 | hypothetical phage protein |
| BPSL0132 | hypothetical phage protein |
| BPSL0133 | hypothetical phage protein |
| BPSL0134 | putative phage-encoded membrane protein |
| BPSL0135 | conserved hypothetical phage protein |
| BPSL0136 | hypothetical phage protein |
| BPSL0137 | hypothetical phage protein |
| BPSL0138 | putative phage protein |
| BPSL0139 | putative phage DNA-binding protein |
| BPSL0140 | hypothetical phage protein |
| BPSL0141 | putative phage DNA-binding protein |
| BPSL0142 | putative phage-encoded membrane protein |
| BPSL0143 | hypothetical phage protein |
| BPSL0144 | putative phage protein |
| BPSL0145 | putative phage protein |
| BPSL0146 | putative phage-encoded membrane protein |
| BPSL0147 | putative phage protein |
| BPSL0148 | putative phage protein |
| BPSL0149 | phage major tail tube protein |
| BPSL0150 | phage major tail sheath protein |
| BPSL0151 | putative phage tail fiber assembly protein |
| BPSL0152 | phage-related tail fiber protein |
| BPSL0153 | putative phage protein |
| BPSL0154 | phage baseplate assembly protein |
| BPSL0155 | phage baseplate assembly protein |
| BPSL0156 | phage baseplate assembly protein |
| BPSL0157 | phage-encoded modification methylase |
| BPSL0158 | putative phage protein |
| BPSL0159 | phage tail completion protein |
| BPSL0160 | phage tail completion protein |
| BPSL0161 | putative phage protein |
| BPSL0162 | putative phage-encoded lipoprotein |
| BPSL0163 | putative phage-encoded peptidoglycan binding protein |
| BPSL0164 | putative phage-encoded membrane protein |
| BPSL0165 | putative phage-encoded membrane protein |
| BPSL0166 | phage tail protein |
| BPSL0167 | hypothetical phage protein |
| BPSL0168 | phage head completion/stabilization protein |
| BPSL0169 | phage terminase, endonuclease subunit |
| BPSL0170 | phage major capsid protein precursor |
| BPSL0171 | putative phage capsid scaffolding protein |
| BPSL0172 | phage terminase, ATPase subunit |
| BPSL0173 | putative phage portal vertex protein |
| BPSL0174 | putative phage DNA-binding protein |
| BPSL0175 | conserved hypothetical phage protein |
| BPSL0176 | putative phage-encoded membrane protein |
| **GI 6** |  |
| BPSL1137 | putative nucleotide binding protein |
| BPSL1138 | conserved hypothetical protein |
| BPSL1139 | hypothetical protein |
| BPSL1140 | putative phage portal protein |
| BPSL1141 | hypothetical protein |
| BPSL1142 | putative phage protease |
| BPSL1143 | putative phage terminase |
| BPSL1144 | putative exported protein |
| BPSL1145 | HNH endonuclease family phage protein |
| BPSL1146 | hypothetical protein |
| BPSL1147 | hypothetical protein |
| BPSL1148 | hypothetical protein |
| BPSL1149 | hypothetical protein |
| BPSL1150 | hypothetical protein |
| BPSL1151 | hypothetical protein |
| BPSL1152 | hypothetical protein |
| BPSL1153 | hypothetical protein |
| BPSL1153a | hypothetical protein |
| BPSL1154 | hypothetical protein |
| BPSL1155 | conserved hypothetical protein |
| BPSL1156 | hypothetical protein |
| BPSL1157 | putative phage integrase |
| **GI 9** |  |
| BPSL2568 | hypothetical protein |
| BPSL2569 | putative exported protein |
| BPSL2570 | hypothetical protein |
| BPSL2571 | hypothetical protein |
| BPSL2572 | hypothetical protein |
| BPSL2573 | hypothetical protein |
| BPSL2574 | hypothetical protein |
| BPSL2575 | hypothetical protein |
| BPSL2576 | hypothetical protein |
| BPSL2577 | hypothetical protein |
| BPSL2578 | hypothetical protein |
| BPSL2579 | hypothetical protein |
| BPSL2580 | hypothetical protein |
| BPSL2581 | hypothetical protein |
| BPSL2582 | phage-like regulatory protein |
| BPSL2583 | hypothetical protein |
| BPSL2584 | hypothetical protein |
| BPSL2585 | hypothetical protein |
| BPSL2586 | putative phage integrase |
| **GI 11** |  |
| BPSL3257 | putative plasmid recombinase |
| BPSL3258 | conserved hypothetical protein |
| BPSL3259 | putative plasmid conjugal transfer protein |
| BPSL3260 | conserved hypothetical protein |
| BPSL3261 | hypothetical protein |
| BPSL3262 | plasmid conjugal transfer protein |
| BPSL3263 | putative plasmid conjugal transfer protein |
| BPSL3264 | plasmid conjugal transfer relaxase protein |
| BPSL3265 | putative plasmid conjugal transfer protein |
| BPSL3266 | hypothetical protein |
| BPSL3267 | hypothetical protein |
| BPSL3268 | putative membrane protein |
| BPSL3269 | hypothetical protein |
| BPSL3270 | putative plasmid replication protein |
| **GI 16** |  |
| BPSS2051 | putative DNA-binding protein |
| BPSS2052 | putative two-partner secretion system protein |
| BPSS2053 | putative cell surface haemagluttinin protein |
| BPSS2054 | conserved hypothetical protein |
| BPSS2055 | conserved hypothetical protein |
| BPSS2056 | conserved hypothetical protein |
| BPSS2057 | putative IS element transposase |
| BPSS2058 | putative ATP-binding inner membrane transport protein |
| BPSS2059 | putative endoribonuclease |
| BPSS2060 | L-asparaginase |
| BPSS2061 | putative DNA-binding protein |
| BPSS2061a | putative transposase (fragment) |
| BPSS2062 | acetyltransferase (GNAT) family protein |
| BPSS2063 | hypothetical protein |
| BPSS2063a | hypothetical protein |
| BPSS2064 | putative porin protein |
| BPSS2065 | putative fatty aldehyde dehydrogenase |
| BPSS2066 | conserved hypothetical protein |
| BPSS2067 | putative aldose 1-epimerase |
| BPSS2068 | short chain dehydrogenase |
| BPSS2069 | ABC transporter, ATP-binding protein |
| BPSS2070 | branched-chain amino acid transport system permease |
| BPSS2071 | putative exported protein |
| BPSS2072 | mandelate racemase / muconate lactonizing enzyme |
| BPSS2073 | GntR family regulator protein |
| BPSS2074 | senescence marker protein-30 (SMP-30) family protein |
| BPSS2074a | conserved hypothetical protein (fragment) |
| BPSS2075 | conserved hypothetical protein |
| BPSS2076 | transposase IS66 family protein (pseudogene) |
| BPSS2078 | putative IS element protein |
| BPSS2079 | putative transposase |
| BPSS2080 | hypothetical protein |
| BPSS2081 | putative alpha-galactosidase |
| BPSS2082 | putative ABC transporter system permease |
| BPSS2083 | putative ABC transporter, permease protein |
| BPSS2084 | putative extracellular solute-binding protein |
| BPSS2085 | putative ABC transport system, ATP-binding protein |
| BPSS2086 | hypothetical protein |
| BPSS2087 | LacI family regulatory protein |
| BPSS2088 | transposase (fragment) |
| BPSS2089 | putative exported protein |
| BPSS2090 | putative DNA-binding protein |
