# Supplementary figures and images for: Burkholderia pseudomallei genome plasticity associated with genomic island variation
Source: BMC Genomics. 2008 Apr 25;9:190. doi: 10.1186/1471-2164-9-190 (PMC2386483; doi:10.1186/1471-2164-9-190)

K96243

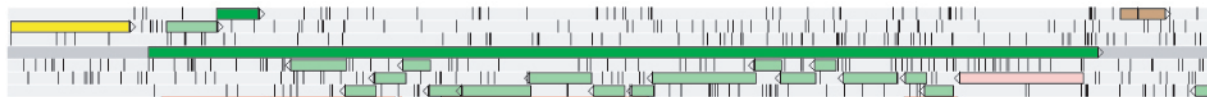

1106a

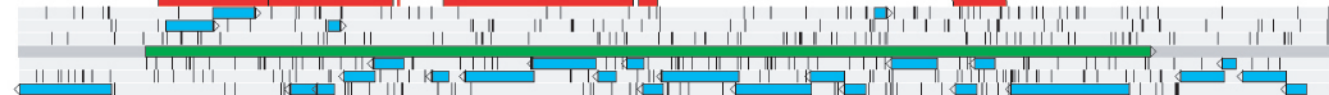

Supplement: Additional file 3 — Comparison of GI 9 in B. pseudomallei strain K96243 with a similar island in B. pseudomallei strain 1106a. The result of a BLASTN comparison of the GI 9 from K96243 (top) with a similar island present at an alternative locus in 1106a (bottom) is displayed using the Artemis Comparison Tool (ACT). The red bars represent similarity matches identified by BLASTN analysis. Horizontal green bars mark the extent of the island in each sequence. [file 1471-2164-9-190-S3.pdf]
